# A Web-Based Peer-Modeling Intervention Aimed at Lifestyle Changes in Patients With Coronary Heart Disease and Chronic Back Pain: Sequential Controlled Trial

**DOI:** 10.2196/jmir.3434

**Published:** 2014-07-23

**Authors:** Rebecca Schweier, Matthias Romppel, Cynthia Richter, Eike Hoberg, Harry Hahmann, Inge Scherwinski, Gregor Kosmützky, Gesine Grande

**Affiliations:** ^1^Faculty of Architecture and Social SciencesUniversity of Applied Sciences Leipzig (HTWK Leipzig)LeipzigGermany; ^2^Institute for Public Health and Nursing Research, Department Prevention and Health PromotionFaculty 11: Human and Health SciencesUniversity of BremenBremenGermany; ^3^Mühlenbergklinik Holsteinische SchweizBad Malente-GremsmühlenGermany; ^4^Klinik SchwabenlandIsny-NeutrauchburgGermany; ^5^MEDIAN Klinik Bad LausickBad LausickGermany; ^6^Reha-Zentrum Bad Driburg – Klinik BerlinBad DriburgGermany

**Keywords:** coronary artery disease, lifestyle, health behavior, back pain, personal narratives as topic, Internet, diet, exercise, Web-based intervention

## Abstract

**Background:**

Traditional secondary prevention programs often fail to produce sustainable behavioral changes in everyday life. Peer-modeling interventions and integration of peer experiences in health education are a promising way to improve long-term effects in behavior modification. However, effects of peer support modeling on behavioral change have not been evaluated yet. Therefore, we implemented and evaluated a website featuring patient narratives about successful lifestyle changes.

**Objective:**

Our aim is to examine the effects of using Web-based patient narratives about successful lifestyle change on improvements in physical activity and eating behavior for patients with coronary heart disease and chronic back pain 3 months after participation in a rehabilitation program.

**Methods:**

The lebensstil-aendern (“lifestyle-change”) website is a nonrestricted, no-cost, German language website that provides more than 1000 video, audio, and text clips from interviews with people with coronary heart disease and chronic back pain. To test efficacy, we conducted a sequential controlled trial and recruited patients with coronary heart disease and chronic back pain from 7 inpatient rehabilitation centers in Germany. The intervention group attended a presentation on the website; the control group did not. Physical activity and eating behavior were assessed by questionnaire during the rehabilitation program and 12 weeks later. Analyses were conducted based on an intention-to-treat and an as-treated protocol.

**Results:**

A total of 699 patients were enrolled and 571 cases were included in the analyses (control: n=313, intervention: n=258; female: 51.1%, 292/571; age: mean 53.2, SD 8.6 years; chronic back pain: 62.5%, 357/571). Website usage in the intervention group was 46.1% (119/258). In total, 141 trial participants used the website. Independent *t* tests based on the intention-to-treat protocol only demonstrated nonsignificant trends in behavioral change related to physical activity and eating behavior. Multivariate regression analyses confirmed belonging to the intervention group was an independent predictor of self-reported improvements in physical activity regularity (β=.09, *P*=.03) and using less fat for cooking (β=.09, *P*=.04). In independent *t* tests based on the as-treated protocol, website use was associated with higher self-reported improvements in integrating physical activity into daily routine (*d*=0.22, *P*=.02), in physical activity regularity (*d*=0.23, *P*=.02), and in using less fat for cooking (*d*=0.21, *P*=.03). Multivariate regression analyses revealed that using the website at least 3 times was the only factor associated with improved lifestyle behaviors.

**Conclusions:**

Usage of the lebensstil-aendern website corresponds to more positive lifestyle changes. However, as-treated analyses do not allow for differentiating between causal effects and selection bias. Despite these limitations, the trial indicates that more than occasional website usage is necessary to reach dose-response efficacy. Therefore, future studies should concentrate on strategies to improve adherence to Web-based interventions and to encourage more frequent usage of these programs.

##  Introduction

Noncommunicable and chronic diseases, such as coronary heart disease or chronic back pain, create a substantial personal and public burden globally. Diseases of the circulatory system account for 35% of deaths in Europe [1]. Chronic back pain is one of the most frequently occurring diseases of modern civilization; estimated 1-month prevalence is more than 20% globally [2]. Lifestyle-related risk factors, such as physical inactivity and unhealthy eating, are associated with a higher risk for these chronic diseases [3-7]. Thus, lifestyle change is a central aim in secondary prevention. Traditional secondary prevention programs, such as inpatient rehabilitation programs or back therapy training, have proved to be successful in the short term, but they often fail to generate sustainable behavioral changes in everyday life [8,9].

Peer-modeling interventions and integration of peer support and peer experiences in health education are a promising way to improve long-term effects in disease management [10-13]. According to social cognitive theory [14], complex behavioral patterns can be learned by watching others. In research concerning patients’ expectations and information-seeking behavior, patients often report a special interest in patient narratives and peer support [15-17]. Peer support is shown to be effective in improving self-efficacy and recovery from surgery in patients with heart diseases [18,19] as well as reducing disability and pain in chronic pain patients [20]. Interventions featuring patient narratives have a positive effect on cancer screening decisions [21] and self-care in diabetes patients [22,23].

Patient narratives about illness experiences can be found on various Web forums, such as video and social networking websites (eg, YouTube and Facebook), and on publicly funded websites such as the healthtalkonline website (formerly DIPEx) [24]. However, empirical data on the impact of patient narratives and peer modeling on lifestyle behavior modification are still lacking. Therefore, we implemented and subsequently evaluated a website featuring patient narratives about successful lifestyle changes. Our objective was to examine whether the use of this website [25] contributes to improvements in physical activity and eating behavior in chronically ill coronary heart disease and back pain patients.

## Methods

### The Website

#### Overview

The website lebensstil-aendern (“lifestyle-change”) is a nonrestricted, no-cost, German language website that provides more than 1000 video, audio, and text clips from interviews with patients living with coronary heart disease and chronic back pain. The website has been online since November 2011 and is certified with the Health on the Net Foundation Code of Conduct (HONcode), a certificate addressing reliability and trustworthiness of medical information on the Internet [26]. Details on implementation, usability evaluation, and usage statistics are reported elsewhere [27].

#### The Patient Narratives

We interviewed 39 people with coronary heart disease and 27 with chronic back pain who reported that they had successfully modified their behavior in at least 1 lifestyle domain for more than 6 months. These problem-centered interviews [28] focused on strategies and barriers in maintaining a healthy lifestyle. Interviewees were recruited across Germany through media coverage, information events, flyers, word-of-mouth recommendations, and social media.

The interviews lasted between 1 to 3 hours. We extracted short clips addressing different aspects of lifestyle modification. All clips are provided with text and may also contain a video or audio clip depending on the interviewees’ preferences. Before publication, patients were contacted again to decide which clips should be published, whether statements should be removed, and if they wanted to use a pseudonym or their real name. For quality assessment and to avoid potentially harmful suggestions, experienced cardiologists and orthopedists reviewed all patient statements before publication.

#### Website Structure and Content

**Figure 1:**
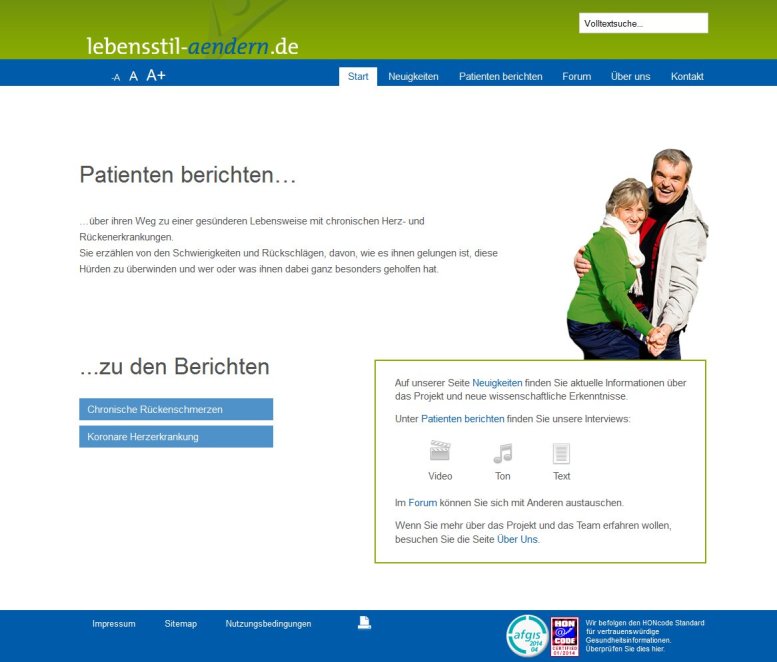
Screenshot of the lebensstil-aendern home page.

The website is structured using a horizontal menu with links to the home page, a news page, the patient narratives, a forum, background information, and a contact form (see Figure 1).

**Figure 2:**
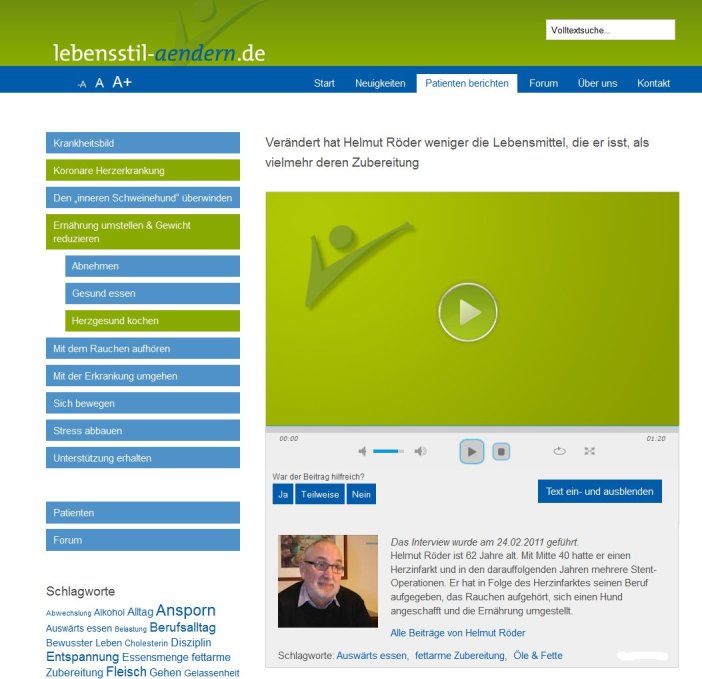
Screenshot of a video subpage from the coronary heart disease module. On the left side is the vertical menu with the tag cloud beneath. Below the video player are buttons for evaluation and short information text about the patient’s health condition.

The news page is updated several times a month by the project team and provides news about recent research results and announcements of new patient narratives, recipes, and project-related updates. The patient narratives are divided into 2 indication-specific modules and structured using a vertical menu with the following categories: overcoming your “weaker self”, getting active, eating healthier, reducing stress, getting support, dealing with the disease, quitting smoking (only in the coronary heart disease module), and keeping the spine in mind (only in the chronic back pain module). In addition to the menu, suitable clips can be found via a filter for age and sex, a tag cloud, searching for keywords, or through overview pages for each patient. Users can comment on single clips and evaluate them (see Figure 2).

The forum contains the same categories as the patient narratives. Posts are accessible for everyone, but users must be registered and logged in to post to the forum.

### Recruitment

**Figure 3:**
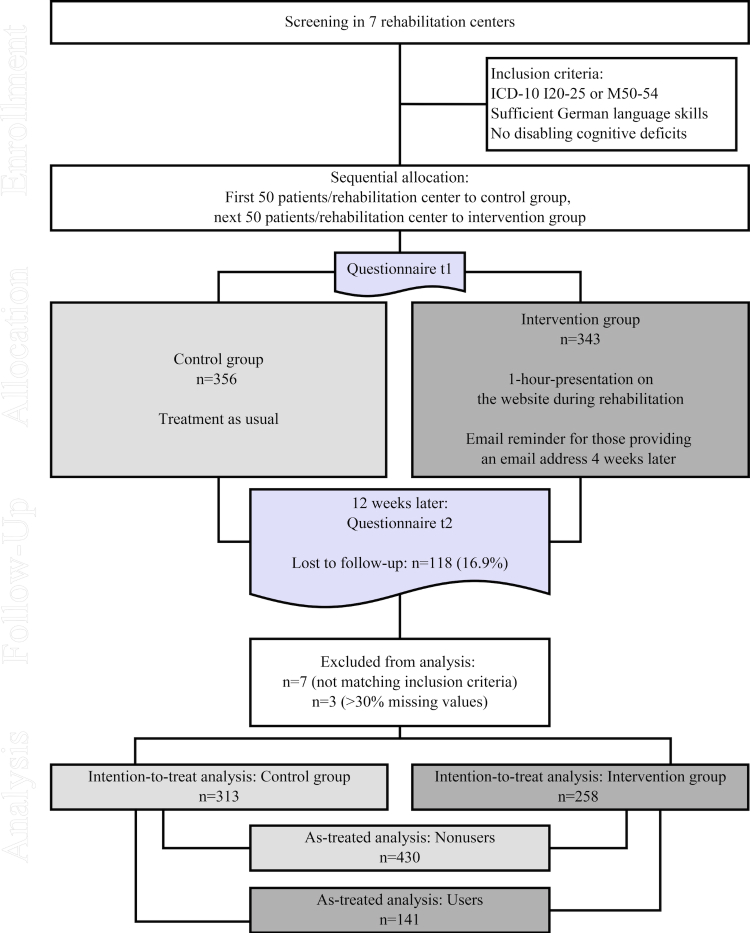
Flowchart of study participants.

We recruited patients in 7 inpatient rehabilitation centers from September 2012 to August 2013. Patients had to be diagnosed with coronary heart disease (International Statistical Classification of Diseases and Related Health Problems, ICD-10 I20-25) or chronic back pain (ICD-10 M50-54), have sufficient German language skills, and have no disabling cognitive deficits. All patients meeting the inclusion criteria were enrolled and sequentially assigned to the control group and intervention group, respectively (see Figure 3). Sequential allocation was chosen to avoid contamination between groups. The study protocol was approved by the Ethics Committee of the Faculty of Medicine, Leipzig University (reference number: 301-10-04102010).

### Intervention

Patients in the intervention group were invited to join a presentation about the website. During the 1-hour presentation, the project team introduced the aims and scopes of the project and provided information on how to find suitable content on the website. They also discussed how to register and post on the forum, and addressed issues of data protection, including anonymity. To give the participants an idea of the website content, 1 to 3 patient narrative videos were shown. To further reduce barriers to using the website, the intervention group received a detailed printed manual and was encouraged to contact the project team in case of problems or questions. Those participants who provided an email address received an email reminder with the link to the website 4 weeks after the presentation. Patients in the control group received no intervention.

### Measures

Both the intervention and control groups completed a questionnaire at some point during participation in a rehabilitation program. Questions addressed sociodemographic characteristics (eg, age, sex, education, and income), diagnosis, body mass index (BMI), and baseline behavior for physical activity and eating routine. The latter 2 were measured on a numerical scale of 0 to 10 asking about the frequency of exercise and the attention paid to a healthy diet. The follow-up questionnaire also included 2 questions about exercise frequency and attention paid to healthy diet. Additionally, patients were asked to rate, on a 5-point Likert scale ranging from slightly deteriorated (–1) to improved substantially (3), their improvements in physical activity (frequency, regularity, integration in daily routine) and eating behavior (eating more healthy foods, avoiding unhealthy foods, eating smaller portions, using less fat for cooking). Lastly, usage of and satisfaction with the website were assessed.

### Statistical Analysis

Statistical analysis was conducted using SPSS Statistics version 21 (IBM Corp, Armonk, NY, USA). Uncertainties during data entry (eg, the participants’ handwriting was difficult to decipher or more than 1 box marked as an answer) were discussed by the team and resolved by consensus. Seven participants were excluded for not matching the ICD-10 diagnoses I20-25 or M50-54 and all 118 dropout cases and 3 other cases were excluded for having more than 30% missing data. Missing data in the remaining sample were estimated using multiple imputations under a fully conditional specification and 10 iterations. Because 41.3% (236/571) cases had missing values in any of the variables to be imputed, we imputed 60 datasets [29]. For evaluation, we compared groups by using independent *t* tests and chi-square tests and calculated Cohen’s *d*, *P* values, and 95% confidence intervals for the mean difference between groups (Δ). For regression analyses, regression weights (B) plus their 95% confidence intervals and beta values (standardized regression weights) are reported.

For estimating improvements in exercise frequency and attention paid to a healthy diet, we calculated mean differences (Δ) between the 2 measure points and compared these mean differences in independent *t* tests. Results are reported both for an intention-to-treat analysis (intervention group vs control group) as well as for an as-treated analysis (website users vs website nonusers). For the regression analyses on the various outcomes in the intention-to-treat analysis, we included intervention group, indication of coronary heart disease, male sex, age per decade, more than 10 years of education, income per €200, BMI, and baseline behavior (exercise frequency for physical activity items and attention toward a healthy diet for eating behavior items). For the regression analyses on the various outcomes in the as-treated analysis, we replaced the intervention group variable with frequent website usage (at least 3 times) and occasional website usage (once or twice).

## Results

### Dropout Analysis

A total of 699 participants filled out the first questionnaire during rehabilitation. During follow-up, 118 patients (16.8%) dropped out. Dropout was higher among patients with coronary heart disease (21.5% vs 14.1%, χ^2^_1_=6.3, *P*=.01) and in the intervention group (23.2% vs 11.1%, χ^2^_1_=18.1, *P*<.001). On average, those who dropped out were 1.9 years younger (*t*_685_=2.11, *P*=.04) and had a €189 lower income (*t*_623_=2.74, *P*=.006).

### Website Usage

In total, 24 of 313 control group participants (7.7%) and 164 of 258 intervention group participants (63.6%) claimed to know the website. Nevertheless, approximately one-quarter of these patients (46/188, 24.5%) never visited the website. Overall, 119 of 258 in the intervention group (46.1%) and 22 of 313 in the control group (7.0%) visited the website; 1 participant (0.7%) stated he used the website 3 to 4 times a week, 6 participants (4.3%) stated they used it once or twice a week, 60 (42.6%) used it once or twice a month, and 74 (52.5%) accessed the website at least once.

### Intention-to-Treat Analysis

Participants had a mean age of 53.2 years (SD 8.6), 51.1% (292/571) were women, and 62.5% (357/571) had been diagnosed with chronic back pain (Table 1). Intervention group participants had higher levels of education than control group participants. Income was distributed equally in both samples, but it was slightly lower than the average German net equivalent income of €1835 per month in 2012 [30]. Participants had a preobese BMI of 28.1 kg/m^2^ (SD 5.2), much higher than the German average (25.7 kg/m^2^ in 2009 [31]).

There were no significant differences in outcome variables between the groups (see Table 2). The intervention group showed a tendency to improve the regularity of their physical activity more than the control group but this did not meet statistical significance in *t* tests. In the regression analysis, belonging to the intervention group emerged as an independent predictor for more recognizable improvements in regularity of physical activity, including younger age, higher income, lower baseline exercise frequency, and prevalence of coronary heart disease compared to chronic back pain (see Table 3). In addition to this, we found a trend in *t* tests in favor of the intervention group for using less fat for cooking. In the regression analysis, belonging to the intervention group also emerged as an independent predictor, accompanied by independent effects of prevalence of coronary heart disease compared to chronic back pain, lower education, and higher BMI.

**Table 1:** Baseline characteristics—intention-to-treat analysis.

Variable		Control group (n=313)	Intervention group (n=258)	Total (n=571)	*t*_569_	χ^2^_1_	*P*
Age (years), mean (SD)		52.7 (8.1)	53.8 (9.2)	53.2 (8.6)	–1.52		.13
**Sex, n (%)**							
	Female	170 (54.3)	122 (47.3)	292 (51.1)			
	Male	143 (45.7)	136 (52.7)	279 (48.9)		0.2	.69
**Indication, n (%)**							
	Coronary heart disease	115 (36.7)	99 (38.4)	214 (37.5)			
	Chronic back pain	198 (63.3)	159 (61.6)	357 (62.5)		2.8	.10
**Education, n (%)**							
	≤10 years of school	236 (75.5)	172 (66.8)	408 (71.5)			
	>10 years of school	77 (24.5)	86 (33.2)	163 (28.5)		4.9	.03
Net equivalent income per month (€), mean (SD)		1679 (621)	1713 (633)	1695 (627)	–0.62		.56
BMI (kg/m^2^), mean (SD)		28.3 (5.3)	27.9 (5.2)	28.1 (5.2)	0.82		.41
Exercise frequency (scale 0-10), mean (SD)		3.89 (2.88)	4.27 (2.81)	4.06 (2.85)	–1.61		.11
Attention paid to a healthy diet (scale 0-10), mean (SD)		5.61 (2.37)	5.59 (2.60)	5.60 (2.47)	0.10		.92

**Table 2:** Independent *t* tests—intention-to-treat analysis.

Dependent variable		Control group (n=313)	Intervention group (n=258)	Mean difference	*t*_569_	95% CI	Cohen’s *d*
**Physical activity**							
	Frequency of doing exercise, Δ (t2–t1)	0.77	0.51	–0.26	3.17	–0.79, 0.27	–0.08
	Improvements in physical activity frequency, mean	1.26	1.33	0.07	0.92	–0.09, 0.23	0.08
	Improvements in physical activity regularity, mean	1.19	1.33	0.14	0.98	–0.03, 0.30	0.14
	Improvements in physical activity in daily routine, mean	1.23	1.31	0.08	0.97	–0.08, 0.24	0.08
**Eating behavior**							
	Attention paid to a healthy diet, Δ (t2–t1)	1.10	1.20	0.11	2.34	–0.29, 0.51	0.04
	Improvements in eating more healthy foods , mean	1.31	1.32	0.01	0.88	–0.14, 0.17	0.01
	Improvements in eating less unhealthy foods, mean	1.32	1.39	0.08	0.95	–0.09, 0.24	0.08
	Improvements in eating smaller portions, mean	1.08	1.10	0.03	0.89	–0.13, 0.18	0.03
	Improvements in using less fat for cooking, mean	1.17	1.30	0.13	0.88	–0.03, 0.30	0.14

**Table 3:** Multivariate linear regression—intention-to-treat analysis. Each line starting with a dependent variable contains information from one regression analysis (N=571) and provides model fit (*R* ^2^), results for the variable group (regardless of significance), and those variables which were demonstrated to be significant independent predictors in the model.

Dependent variable		*R* ^*2*^	Group		Further independent predictors		
			B (95% CI)	β	Variable	B (95% CI)	β
**Physical activity**							
	Frequency of doing exercise, Δ (t2–t1)	.42	0.01 (–0.40, 0.42)	.001	Indication	0.63 (0.12, 1.13)	.10
					Age	–0.40 (–0.65, –0.14)	–.11
					Baseline behavior	–0.70 (–0.78, –0.63)	–.63
	Improvements in physical activity frequency	.06	0.10 (–0.06, 0.26)	.05			
					Age	–0.18 (–0.28, –0.09)	–.17
					Income	0.04 (0.01, 0.07)	.13
					Baseline behavior	–0.03 (–0.06, 0.00)	–.09
	Improvements in physical activity regularity	.06	0.18 (0.02, 0.34)	.09	Indication	0.21 (0.01, 0.42)	.11
					Age	–0.18 (–0.28, –0.08)	–.16
					Baseline behavior	–0.03 (–0.06, 0.00)	–.10
	Improvements in physical activity in daily routine	.06	0.11 (–0.05, 0.27)	.08	Age	–0.13 (–0.23, –0.03)	–.12
					Education	–0.28 (–0.46, –0.09)	–.13
**Eating behavior**							
	Attention paid to a healthy diet, Δ (t2–t1)	.44	0.12 (–0.19, 0.42)	.02	Baseline behavior	–0.63 (–0.70, –0.57)	–.65
	Improvements in eating more healthy foods	.08	0.03 (–0.12, 0.18}	.02	BMI	0.04 (0.02, 0.05)	.20
	Improvements in eating less unhealthy foods	.09	0.08 (–0.08, 0.23)	.04	Indication	0.29 (0.09, 0.48)	.15
					BMI	0.03 (0.02, 0.05)	.18
	Improvements in eating smaller portions	.09	0.05 (–0.10, 0.20)	.03	BMI	0.05 (0.04, 0.07)	.29
	Improvements in using less fat for cooking	.07	0.16 (0.01, 0.31)	.09	Indication	0.20 (0.01, 0.39)	0.11
					Education	–0.22 (–0.40, –0.05)	–.11
					BMI	0.03 (0.02, 0.04)	.19

### As-Treated Analysis

Users had higher levels of education than nonusers. We did not find any significant differences in baseline variables between nonusers and users in the as-treated analysis (see Table 4).

**Table 4:** Baseline characteristics—as-treated analysis.

Variable		Nonusers (n=430)	Users (n=141)	*t*_569_	χ^2^_1_	*P*
Age (years), mean (SD)		53.5 (8.6)	52.2 (8.4)	–1.56		.12
**Sex, n (%)**						
	Female	217 (50.5)	75 (53.2)			
	Male	213 (49.5)	66 (46.8)		0.3	.57
**Indication, n (%)**						
	Coronary heart disease	165 (38.4)	49 (34.8)			
	Chronic back pain	265 (61.6)	92 (65.2)		0.6	.44
**Education, n (%)**						
	≤10 years of school	317 (73.7)	92 (65.2)			
	>10 years of school	113 (26.3)	49 (34.8)		3.9	.049
Net equivalent income per month (€), mean (SD)		1667 (626)	1780 (622)	–1.81		.07
BMI (kg/m^2^), mean (SD)		27.9 (5.1)	28.8 (5.5)	1.72		.09
Frequency of doing exercise, (scale 0-10), mean (SD)		4.09 (2.89)	3.98 (2.74)	–0.38		.71
Attention paid to a healthy diet (scale 0-10), mean (SD)		5.59 (2.45)	5.62 (2.54)	0.14		.89

Website use was associated with larger increases in physical activity: users were more successful in integrating physical activity into their daily routine and in improving their physical activity regularity. We did not find a significantly higher mean difference in the frequency of doing exercise between baseline and follow-up 12 weeks later.

Website use was not associated with higher mean differences in the attention paid to a healthy diet, but it was with more success in dietary behaviors (ie, using less fat for cooking) (Table 5).

**Table 5:** Independent *t* tests—as-treated analysis.

Dependent variable		Nonusers (n=430)	Users (n=141)	Mean difference	*t*_569_	95% CI	Cohen’s *d*
**Physical activity**							
	Frequency of doing exercise, Δ (t2–t1)	0.60	0.81	0.21	0.67	–0.40, 0.82	0.07
	Improvements in physical activity frequency, mean	1.26	1.40	0.14	1.56	–0.04, 0.32	0.15
	Improvements in physical activity regularity, mean	1.20	1.42	0.22	2.33	0.04, 0.41	0.23
	Improvements in physical activity in daily routine, mean	1.21	1.43	0.21	2.24	0.03, 0.40	0.22
**Eating behavior**							
	Attention paid to a healthy diet, Δ (t2–t1)	1.09	1.32	0.23	0.99	–0.23, 0.69	0.10
	Improvements in eating more healthy foods, mean	1.28	1.43	0.15	1.64	–0.03, 0.33	0.16
	Improvements in eating less unhealthy foods, mean	1.31	1.47	0.16	1.65	–0.03, 0.34	0.16
	Improvements in eating smaller portions, mean	1.04	1.22	0.17	1.91	–0.05, 0.35	0.19
	Improvements in using less fat for cooking, mean	1.18	1.38	0.20	2.17	0.02, 0.38	0.21

In the multivariate regression analyses (Table 6), we found small independent effects of frequent website use on improvements in physical activity regularity and improvements in integration of physical activity into daily routine. We also found small effects of frequent website use on all 5 variables measuring eating behavior—frequent website use was an independent predictor for increased attention paid to a healthy diet, improved use of healthy and avoidance of unhealthy foods, improvements in eating smaller portions, and reduced use of fat for cooking.

**Table 6:** Multivariate linear regression—as-treated analysis. Each line starting with a dependent variable contains information from one regression analysis (N=571) and provides model fit (*R* ^2^), results for the variables occasional website usage and frequent website usage (regardless of significance), and those variables which were demonstrated to be significant independent predictors in the model.

Dependent variable		*R* ^*2*^	Occasional website usage		Frequent website usage		Further independent predictors		
			B (95% CI)	β	B (95% CI)	β	Variable	B (95% CI)	β
**Physical activity**									
	Frequency of doing exercise, Δ (t2–t1)	.42	–0.13 (–0.74, 0.49)	–.01	0.28 (–0.36, 0.92)	.03	Indication	0.63 (0.13, 1.14)	.10
							Age	–0.41 (–0.66, –0.15)	–.11
							Baseline behavior	–0.70 (–0.77, –0.62)	–.63
	Improvements in physical activity frequency	.06	–0.005 (–0.23, 0.22)	–.002	0.21 (–0.03, 0.45)	.07	Age	–0.18 (0.28, 0.09)	–.17
							Income	0.04 (0.01, 0.07)	.13
	Improvements in physical activity regularity	.06	0.12 (–0.12, 0.36)	.04	0.27 (0.02, 0.51)	.09	Indication	0.21 (0.01, 0.41)	.11
							Age	–0.18 (–0.28, –0.08)	–.16
							Baseline behavior	–0.03 (–0.06, 0.00)	–.09
	Improvements in physical activity in daily routine	.07	0.06 (–0.17, 0.30)	.02	0.39 (0.14, 0.63)	.13	Age	–0.13 (–0.23, –0.03)	–.12
							Education	–0.27 (–0.46, –0.09)	–.13
**Eating behavior**									
	Attention paid, a healthy diet, Δ (t2–t1)	.43	–0.14 (–0.59, 0.31)	–.02	0.73 (0.26, 1.21)	.10	Baseline behavior	–0.64 (–0.71, –0.58)	–.66
	Improvements in eating more healthy foods	.09	–0.06 (–0.28, 0.17)	–.02	0.36 (0.12, 0.59)	.12	BMI	0.03 (0.02, 0.05)	.19
							Baseline behavior	–0.04 (–0.07, 0.00)	–.10
	Improvements in eating less unhealthy foods	.10	0.04 (–0.20, 0.27)	.01	0.32 (0.08, 0.56)	.11	Indication	0.29 (0.10, 0.48)	.15
							BMI	0.03 (0.02, 0.05)	.17
	Improvements in eating smaller portions	.10	0.03 (–0.20, 0.25)	.01	0.28 (0.05, 0.51)	.10	BMI	0.05 (0.03, 0.06)	.28
	Improvements in using less fat for cooking	.08	0.02 (–0.21, 0.25)	.01	0.40 (0.17, 0.64)	.14	Indication	0.20 (0.01, 0.39)	.11
							Education	–0.21 (–0.39, –0.03)	–.10
							BMI	0.03 (0.02, 0.05)	.18

## Discussion

### Principal Results

The aim of this study was to examine the effects of a Web-based intervention featuring patient narratives about successful lifestyle changes on physical activity and eating behavior for chronically ill coronary heart disease and back pain patients. We conducted a sequential controlled trial in which the intervention group participated in a presentation about the website during rehabilitation.

In the intention-to-treat analysis, there were no significant effects of the intervention in the bivariate analyses and only small interventional effects on physical activity regularity and on using less fat for cooking in the multivariate analyses. However, all but 1 (frequency of doing exercise) of the measured outcome variables showed positive tendencies in the expected direction. Comparing website users to nonusers in the as-treated analysis, we found an association between website usage and improvements in most physical activity and dietary behavior outcome variables. Multivariate regression analyses revealed that this association persists only in conjunction with frequent website usage (defined as having visited the website at least 3 times) not with occasional website usage.

### Implications and Future Research

A larger sample size, associated with a sufficiently large power, may have ensured the detection of even small intervention effects. In any case, effect sizes were consistently small and demand a closer look at possible reasons. First, the small intervention effects in the intention-to-treat analysis can be explained in part by the low usage of the website because less than half of the intervention group visited the website. The problem of low usage rates is well known and an often cited problem in trials on Web-based interventions [32-35]. Some personal factors, such as young age, male gender, low income, and low levels of education, are associated with lower rates of preventive service utilization in general [36], further increasing the socially determined health-related disadvantages. In our study, only a lower educational level was related to lower rates of website usage. Thus, other groups who are often difficult to reach seem to benefit from the website.

In addition to the low utilization rates, the intervention dose was probably too low to result in behavioral modifications for most participants. The results of the as-treated analysis indicate that more than occasional website usage is necessary to reach dose-response efficacy. This finding is consistent with results from other studies [37-39] that also found a positive relation between the number of log-ins and outcomes. In these studies, the necessary number of log-ins also was not reached among all users.

Finally, although the integration of peer experiences is seen as promising in health education and disease management and meets the interest of patients with a chronic condition, the additional inclusion of further behavior modification strategies (eg, individual tailoring or providing feedback) may be required in a peer-modeling approach to show satisfactorily large effects on lifestyle behavior [40].

Future studies should concentrate on strategies to improve adherence to Web-based interventions and especially to induce more frequent usage of these programs. Individually tailored interventions may be a promising approach because these interventions, besides being more efficacious, have also been shown to stimulate more frequent use of Web-based interventions and to increase adherence [21,41].

### Limitations

We were able to assess behavioral change through self-reported nonvalidated measures only, which are subject to response bias. One important aim of rehabilitation programs is to increase awareness for unhealthy lifestyle behavior. Thus, recruiting the participants at this point of time might have led to an overestimation of the 2 baseline variables, which then would have a flattening effect on the mean difference between t2 and t1. Due to time restrictions in the research project, we could only observe the effects of the intervention 3 months after participation in the rehabilitation program. The long-term effects of the intervention remain unclear. Furthermore, we did not control for Internet literacy or Internet usage patterns, which might explain differences in website usage rates and website efficacy.

The as-treated analysis implicates positive results, but does not allow for a causal interpretation because nonmeasured variables associated with more frequent use of the website could account for the improved health behavior or successful patients might tend to more actively seek support and information and use a website such as the lebensstil-aendern website.

For the multivariate regression analyses, model fit was very low. This could either be due to variables accounting for behavioral change but not being included in the model or, considering that all outcome variables were single item variables, unreliable measurements contributing to the large error variances.

### Conclusions

To our knowledge, our study is the first trial to test the efficacy of Web-based patient narratives on behavioral outcomes in coronary heart disease and chronic back pain patients. In our study, patients that use the website more frequently report more favorable health-related behavior and more marked improvements in physical activity and diet. Even if more motivated patients seek support and information more actively, for this patient group in particular, a website such as the lebensstil-aendern website appears to be a helpful tool.

## Abbreviations

BMI: Body mass index
ICD: International Statistical Classification of Diseases and Related Health Problems
